# A Map is More Than a Polygon: Contesting Green Infrastructure in Forested Landscapes in Sweden

**DOI:** 10.1007/s00267-026-02416-1

**Published:** 2026-03-18

**Authors:** Luis Andrés Guillén, Derek Garfield, Vilis Brukas

**Affiliations:** Swedish University of Agricultural Sciences, Southern Swedish Forest Research Centre, Sundsvägen 3, 234 56 Alnarp, Sweden

**Keywords:** Mapping, Forest owners, Green infrastructure, Policy implementation, Planning, Spatial knowledge

## Abstract

Green infrastructure is a novel strategy for spatial prioritisation of forest conservation, where the elaboration of maps is of key importance. Sweden represents an interesting case, where planning for green infrastructure received special attention from governmental authorities. Under Sweden’s liberal forest governance, forest stakeholders (owners, companies and interest groups) acceptance is crucial for implementing novel instruments for landscape management, such as green infrastructure plans. Involving industrial and non-industrial private forest owners is, however, not a simple task. Based on 18 interviews with officials in charge of coordinating the plans at County Administrative Boards, our study aims to explore the challenges in mapping areas of high conservation values (AHCVs), how the coordinators understand the character of these maps as tools for environmental governance, and the resulting conflicts and hindrances to implementation of governmental objectives. Mandated by the Swedish government, green infrastructure plans were intended to gather knowledge, map AHCVs, and list actions to guide stakeholders on how to include green infrastructure in their forest management decisions. We examine how AHCV maps tended to be conceptualised by officials as solely the compilation of new knowledge, allegedly scientific and objective in its nature. However, there was also an acknowledgement that maps can indirectly affect resource distribution for nature conservation or limit forest owners’ decision space. Further, we explore how coordinators adapted to the opposition from forest stakeholders regarding the compilation of AHCV maps. Our discussion centres on how maps, often considered to be pure spatial knowledge, represent a soft policy instrument for environmental governance and resource prioritisation in forestry. Our study underscores how different departure points for knowledge create conflicts and subsequent adaptations in working strategies or stagnation of environmental initiatives.

## Introduction

Historic and contemporary forest management practices have brought unintended consequences, namely the loss of forest biodiversity and habitat connectivity, with the subsequent erosion of the provisioning of regulatory and cultural ecosystem services (Bennett et al., 2009). To address this challenge, Green infrastructure (GI), defined in the European Union Green Infrastructure Strategy as a network of natural and semi-natural habitats that deliver ecosystem services important for biodiversity and human wellbeing (European Commission, 2013), represents a novel nature conservation policy for regional planning through the creation of GI policies (von Post et al., 2023). In this article, we examine the GI policy developments in Sweden, looking specifically at the process of mapping ecologically valuable areas and the associated tensions with stakeholders and the resulting hindrances to GI implementation. Sweden is an interesting case to study GI planning in forest landscapes, being characterised by a vast forest cover, relatively liberal forest regulations, important biodiversity challenges, and a high degree of polarisation regarding forest management versus nature conservation.

GI has been primarily used and studied within city planning, giving way to advancing theoretical and practical frameworks for its analysis (Lafortezza et al., 2013; Pauleit et al., 2019). GI has not been as conspicuous within regional planning in rural settings, despite its relevance to creating functional landscapes (Elbakidze et al., 2017). The Swedish GI policy builds on ecological perspectives and a landscape approach, making spatial knowledge a core element to its implementation (von Post et al., 2023). Ongoing work with GI may also contribute to strengthening the basis for the implementation of the recently adopted EU Nature Restoration Law that aims to restore ecosystems, including areas of high conservation value (AHCVs).

Maps form an integral part of the knowledge system used in the implementation of policies aimed at managing natural resources (Gustafson, 2015; Harley, 2008) and have historically been an important tool for governance (Crampton, 2006; Elden, 2010; Harvey, 2000; Whitehead et al., 2007). Understanding the role of maps as a governance tool is therefore crucial for the successful development of functional GI in forest settings. Land use maps, for instance, do not simply show how land is currently being used, but also indicate potential uses (Whitehead et al., 2007). Therefore, maps play an important role in the governance of natural resources and the prioritisation of the government’s actions. However, governmental agencies’ scope of action is context-dependent, from laissez-faire to heavily regulated governance systems (Lawrence et al., 2020; Nichiforel et al., 2018).

**Fig. 1:**
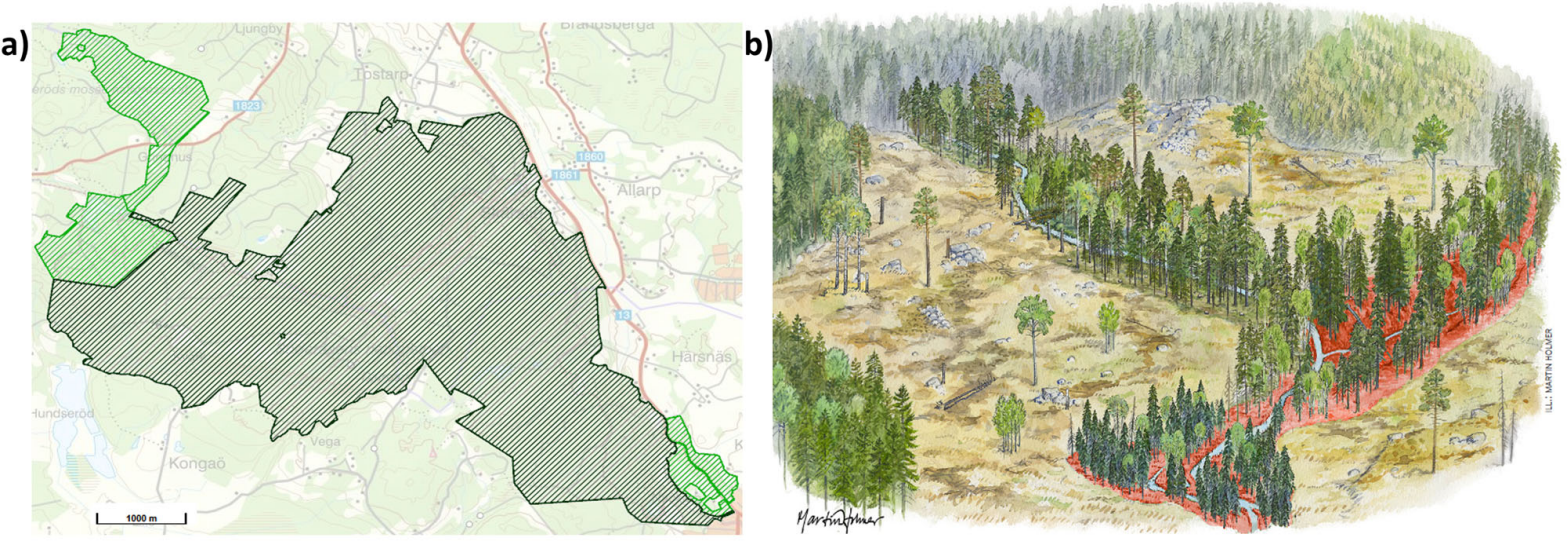
Examples of maps and illustrations as policy instruments: **a** Shows a “hard” policy instrument, where coloured polygons indicate the Söderåsen National Park, Scania (black), and contiguous nature reserve areas (light green); map source: Naturvårdsverket, 2025); **b** shows a soft policy instrument, indicating best management practices for riparian buffers in connection to regeneration harvests, where areas marked with red are recommended to be left unharvested for water protection (Illustration: Martin Holm)

We consider that regulatory and informational policy instruments (Bemelmans-Videc et al., 2017) can be operationalized through maps. In the first case, using maps as a strict regulating instrument tends to be evident: e.g. borders of a national park or a nature reserve clearly indicate that production-oriented forest management activities are forbidden or restricted (Fig. 1a). In the second case, considering maps as informational policy instruments is less obvious when they support “soft law” where no legal mandates or obligations are enforceable, e.g., voluntary set asides, buffers zones or guidelines for best management practices (Fig. 1b), as is commonly the case in the context of achieving broad, non-regulatory policy goals. As part of the soft policy instruments’ toolbox (Lawrence et al., 2020), maps also serve an advisory role for forest owners by guiding their forest management decisions.

Importantly, attempts to implement GI as a form of natural resource governance through mapping have been recently initiated in Sweden (von Post et al., 2023), which permits exploring ongoing adaptations to its implementation. In 2014, the government commissioned each of the 21 Swedish County Administrative Boards (CABs) to develop regional action plans that support biodiversity conservation and ecosystem service provision. GI coordinators were tasked with developing regional GI action plans (from here on GI Plans), which included maps of AHCVs, i.e., landscapes including protected and assumed biodiversity-valuable areas, as well as adjacent land, where biodiversity conservation should receive greater attention (Bakx et al., 2023).

Our paper explores the creation of GI plans in Sweden, with focus on the challenges that mapping nature values entails for the implementation of soft policy instruments, from the perspective of GI coordinators. Our specific objectives were to: (1) explain how maps create tensions with forest stakeholders, (2) examine how maps and mapping AHCVs are understood conceptually and what is their utility, and (3) analyse how tensions lead to hindrances and subsequent adaptation of policy implementation. Our study displays hurdles in the implementation of green infrastructure initiatives and underscores the importance of considering the social construction of maps when undertaking spatial planning initiatives for environmental management.

## Theoretical Underpinnings: Social Construction of Mapping

Maps are a specific form of spatial knowledge – information that gains meaning from human interpretation and processing (Pynnönen, 2020). As with knowledge, maps are socially constructed and do not exist independently from those who create them or use them (Crampton, 2001; Kitchin & Dodge, 2007; Straume, 2014). Considering maps as a social construction means that they no longer need to be thought of as objectively separated from what they represent, their author, or the reader (Crampton, 2001). In that sense, maps represent discourses that contribute to the social construction of the world by organising and displaying space according to a set of social relations (Harley, 2008). When we see maps as a rhetorical tool, the dualism of objective vs. subjective, right vs. wrong, scientific vs. artistic no longer holds (Crampton, 2001). This understanding allows us to focus on how maps are perceived by both the map user and the map creator, as both are valid and represent the experience of their contact with the cartographical object or the ideas that those intend to represent.

The use of maps is widespread in forest sciences and practices. Maps form part of the scientific knowledge systems that are legitimised thanks to the practices, methodologies, infrastructure, and networks that sustain them (Latour, 1988). Contemporarily, expert knowledge is seen as the most valid, even if other types of knowledge – like local or practical knowledge – are also important in forestry (Fortmann & Ballard, 2011; Pynnönen, 2020). However, (natural) scientists and professionals often lack the tools to reflect upon how people understand or interpret knowledge. This lack of reflexivity can create problems when technical knowledge, as represented in a map, does not match how others understand the natural resource or its use (Moran & Rau, 2016; Tsouvalis et al., 2000). In our case, we do not focus on the technical aspects of the maps, but on how they are perceived and what conflicts these perceptions may create. In a complex socio-ecological system such as a forest, a paradigm resting on the view of a measurable world that can be represented spatially can collide with the perception and lived experience of a variety of stakeholders (Gustafson, 2015; Tsouvalis et al., 2000).

Building upon Foucault, maps can be understood as a form of power (Poster 1982, in Harley, 2008; Gustafson, 2015). Creating a map corresponds to creating knowledge within defined structures associated with positions of power (Elden, 2010). Mapping is, therefore, a product of power and a creation of power simultaneously (Kitchin & Dodge, 2007). At the same time, maps function as practical instruments of governance, used by stakeholders to prioritise decisions based on the information that maps transmit (Tsouvalis et al., 2000). Thus, maps both shape understanding of reality and serve as tools for strategic choices. Hence, mapping cannot be considered a neutral recording process (Whitehead et al., 2007) or maps neutral entities (Adams & Sandbrook, 2013). By recognising maps as agents of power, we can better understand their role in strategising environmental management. For our purposes, outcomes of green infrastructure planning are dependent on how maps are conceptualised and how they would be used to define priorities (e.g. areas for conservation, for special management or to be left unmapped for continued production).

## The Swedish Context

### Forest Resources, Ownership and Relevance for Green Infrastructure Planning

More than half of Sweden is forested, representing about 16% (30.3Mha) of the European Union’s (EU) forest (European Commission, 2020), supporting a thriving industry that procures timber from both industrial and non-industrial forest owners. Forest landscapes thus form the primary spatial context within which GI is to be developed and implemented.

Sweden has an heterogeneous landowning structure: the State owns 19% of the land (mainly through the company Sveaskog), private companies own around a quarter of the forestland, mainly in the North, and there are more than 300,000 non-industrial forest owners holding around half of the forestland (Roberge et al., 2020). The ownership structure is particularly relevant for GI, as many ecologically valuable areas would fall on privately owned land; consequently, the acceptance of owners is a major precondition for GI success (von Post et al., 2023).

### Relevant Stakeholders Involved in Forest Governance and GI Processes

Relevant forest stakeholders were those involved in the green infrastructure process at county specific consultations and remittance communications. Given that county specific participation could vary, we describe below the general forest stakeholder constellation participating in GI and in forest governance (for a particular example of county specific participation, please see Karlsson et al., 2024). Swedish forest stakeholders are normally distinguished between either conservation or production interests (Appelstrand, 2012; Eckerberg & Sandström, 2013).

On the production side, key stakeholders include large forest industries, composed of multinational private companies (e.g., SCA, Stora Enso), a large state-owned company (Sveakog), as well as midsize national and regional companies with timber processing or procurement services. Almost a third of Swedish forest owners are members of three forest owners’ associations that offer services and advice, possess industries and engage in lobbying for the sector’s interests (Nilsson et al., 2020). Another key stakeholder is the Federation of Swedish Farmers that seeks to protect the ownership rights of forest owners (Sténs & Mårald, 2020), tending to maintain the relatively strong decision-making and timber extraction rights of Swedish landowners (Nichiforel et al., 2018).

On the nature conservation side, stakeholders include ad hoc and organised local or regional groups, or national organisations with local representation. The Swedish Society for Nature Conservation is a main national NGO when it comes to Swedish forests; international stakeholders, such as the World Wildlife Fund and Greenpeace are also important; other specific interests related to forest protection are represented by various associations, e.g. ornithologists and outdoor recreation enthusiasts. While NGOs do not normally implement conservation measures directly, their advocacy helps the broader governance context in which GI takes place.

Public agencies’ mandates and bureaucracies, due to their history, have been described to be closer to either production or conservation interests (Logmani-Aßmann et al., 2021). For instance, the Swedish Forest Agency (SFA) has long promoted a production-oriented forest model. Its current mandate is to operationalise the Forest Act that equalises production and conservation values. On the environmental side, the County administrative boards (CAB) in each of the 21 counties, acting as local agents of the national government, are in charge of operationalising a large part of the nation’s environmental protection policies with respect to land management, and establishing and managing nature reserves and national parks. Thus, they are the chief public stakeholders in implementing nature conservation, though lacking specific mandates over forestry outside protected areas.

### Liberal Governance and Ensuing Conflicts in Conservation

The Swedish forest and conservation governance context frames the GI policy assemblage described in von Post et al. (2023). Since the 1990’s Swedish forest governance has been characterised by liberal policies, with a relatively low degree of restrictions and the understanding that owners should manage their forest to balance ecological and production values (Appelstrand, 2012).

The Swedish approach to expanding the network of protected areas can be broadly divided into formal and voluntary. Voluntarily protected areas are tightly connected with forest certification, as certifying forest estates is, in principle, voluntary, and at least 5% of productive forests need to be set aside for nature conservation purposes to fulfil certification obligations. Currently, 5.9% (1.39Mha) of productive forest area is voluntarily protected in non-industrial private forests (Swedish Statistical Agency, 2025). Presently, 6.3% (1.44Mha) of productive forest area in Sweden is formally protected, as national parks, nature reserves, and biotope protection areas or areas under nature conservation agreements. Importantly, according to current policies, formally protected areas can only be established having the consent of forest owners who are also fully compensated for the loss of income.

Consistently with Sweden’s liberal forest governance, the use of informational tools, such as GI planning, is of major importance (Brukas & Sallnäs, 2012). Yet, this liberal approach to sectoral deliberation of forest goals does not preclude opposing positions or conflicts in forestry. A well-known case of conflict was the mapping of woodland key habitats inside privately owned forests (Bjärstig et al., 2019; Jakobsson et al., 2021). Inventories of woodland key habitats started in Northern Europe in the 1990’s and have been shown to be of crucial importance as biodiversity hotspots (Timonen et al., 2010). In Sweden, the SFA started carrying out inventories on private land in 1993, which has created conflicts with forest owners (Götmark, 2009). A chief issue in the conflict arises from the sense of an erosion of non-industrial forest owners’ property rights when they felt bypassed by other actors in regard to woodland key habitats, often entailing a significant loss of economic value without compensation (Jakobsson et al., 2021; Sténs & Mårald, 2020). These experiences form an important backdrop for comprehending the responses to GI mapping, which implies the spatial identification of ecologically valuable areas on private land.

#### Green Infrastructure Plans and Maps

GI plans were government mandated and developed regionally by each CAB. Our research was limited to forested landscapes, yet GI plans included all major landscapes present in each county, encompassing e.g. agricultural land, urban green spaces, coastal areas, wetlands and lakes. The intended users of the plans are sectoral stakeholders with decision-making power over land management (e.g., forest managers, landowners, municipal officers), who, thanks to the plans, could make better decisions that consider and support GI (Naturvårdsverket, 2022). Hence, GI planning is intended as both a technical and a governance instrument.

**Fig. 2:**
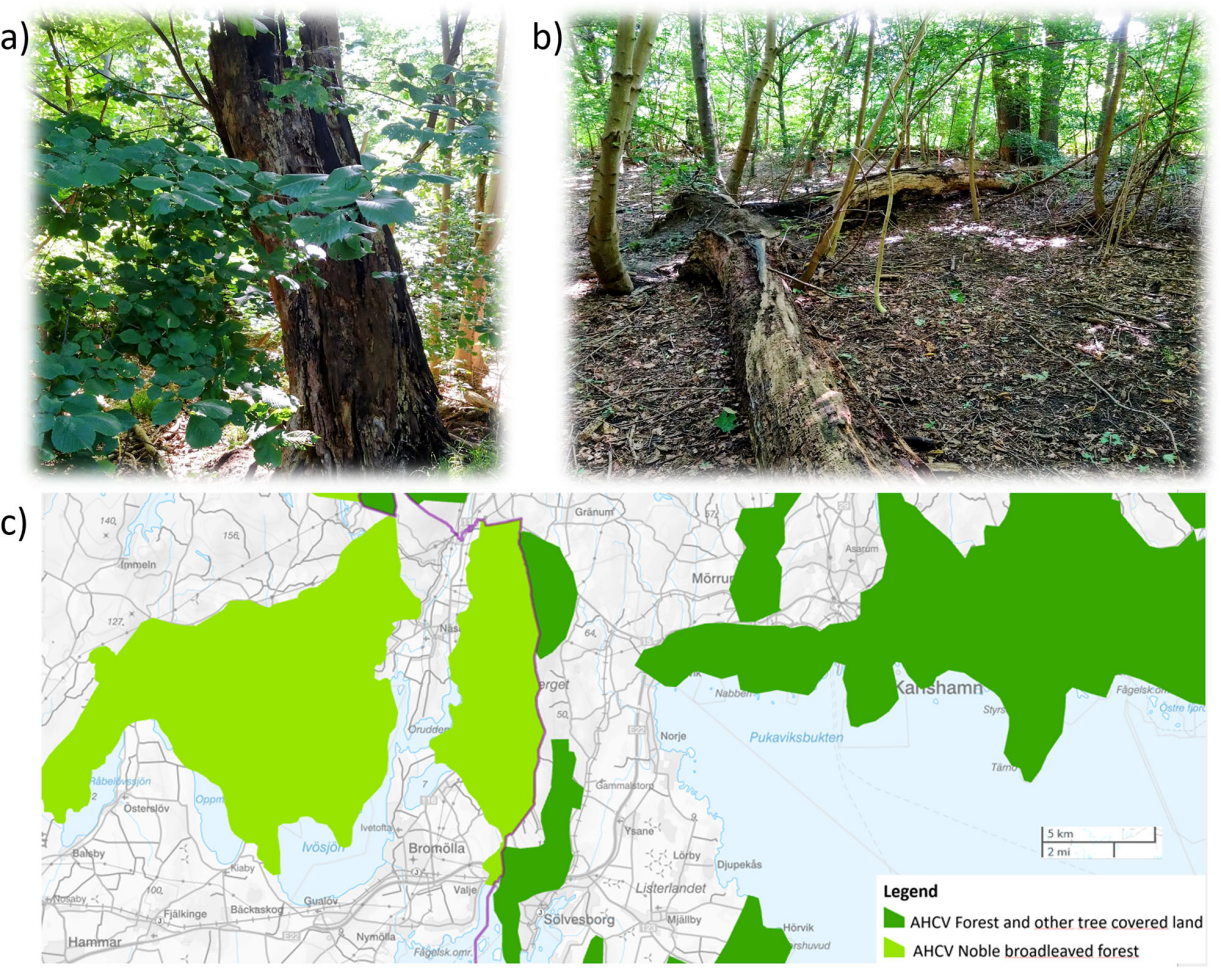
Examples of spatial elements used in GI planning: **a** Standing coarse deadwood as a value element, **b** A forest stand with numerous valuable elements represents a core value area, **c** Screenshot of map of Forest AHCVs around the borders between the counties of Scania and Blekinge. Photos: First author. Map: (Länsstyrelsen, 2024)

A main element of the GI plan is the documentation, compilation and/or mapping of spatially subordinated value elements (*värdeelement* in Swedish), core value zones (*värdekärna*) and areas of high conservation values (AHCVs) (*värdetrakter*) (Table 1 and Fig. 2). These three layers differ greatly in abstraction. Value elements, at the finest scale, represent actual features in the landscape (e.g., large standing deadwood, presence of red-listed species). Upper levels, such as the AHCV, are delineated through Geographical Information System (GIS)-based methods applied by the CABs with the same underlying principles across the country (Naturvårdsverket, 2005). In simple terms, areas of high value were constructed using GIS by compiling and overlaying several spatial datasets representing forest habitat characteristics, species occurrences, and previously identified conservation values, thereby highlighting forest areas with a high density of ecological attributes (Naturvårdsverket, 2005). Here, we do not assess these methods technically or ecologically but rather examine how the maps are conceptualised and perceived in relation to nature conservation actions.

**Table 1:** Definitions of spatial elements used in the GI planning by the county administrative boards (Naturvårdsverket, 2017)

Concept	Scale/Dimension	Example
**Value element:** Elements of positive significance for biodiversity, which describe ecological qualities that are prerequisites for functioning ecosystems, e.g., species, species composition, species complexes, habitats, and functions.	Micro scale. The smallest element used to identify a natural value	Deadwood of large dimensions (Fig. 2a)
**Core value zones:** A coherent natural area that is assessed as having high natural values in terms of its existing natural state. A core value zone normally has a tangible presence of value elements that create conditions for high natural values and rich biodiversity.	Forest stand scale	A forest stand with high structural diversity, presence of coarse deadwood, old age classes, endangered species (Fig. 2b)
**Areas of High Conservation Values (AHCVs):** A landscape section with particularly high ecological conservation values. An AHCV has a significantly higher density of core value zones, including biologically important structures, functions, and processes, than the surrounding landscape.	Landscape scale	Contiguous areas in the landscape (Fig. 2c)

## Methods

The first author interviewed GI coordinators from 18 of the 21 CABs, representing metropolitan areas to rural landscapes, and different vegetation zones, from the boreal North to the nemoral South. GI coordinators were public officers in charge of documentation, preparation, review, and writing of GI action plans for their respective CABs. GI coordinators were professionals with higher education backgrounds in biology, environmental sciences, or ecology. Six coordinators had relatively short experience (<2 years), four had medium experience (2–5 years), and eight had long experience (>5 years) of work in public administration. Twelve of the 18 coordinators were females.

The participants were approached via email, where we presented the aims of the project and asked for the possibility of an interview and their informed consent to participate in the study. After the initial contact, 12 interviews were set up. A second email was sent to coordinators who did not answer, resulting in an additional six interviews. Only one GI coordinator was unresponsive, and two declined to be interviewed since they had not participated actively in the relevant steps of the GI plans due to their short period of employment. Prior to initiating each interview, participants were asked for their consent to be recorded (following GDPR), and we explained that we would take measures to keep their responses anonymous (i.e., using pseudonymisation by not tying specific statements to specific counties), given the limited number of GI coordinators found in the country.

Semi-structured interviews were carried out telematically using either Zoom or Skype, between May and August 2022, with an average duration of 72 min and ranging from 60 to 90 min. Only audio was recorded using a digital recorder. Interviews were semi-structured, inquiring broadly into the GI planning process at each respective CAB and including themes of implementation, participation processes, information and advice, and policy instruments. An advantage of the flexibility and openness of data generation inherent to semi-structured interviews is that it allows for pursuing participants’ ideas and emerging themes, which are important topics to be “discovered” in the data (Bryman, 2016). In our case, the topic of mapping emerged as a very important matter after the first few interviews and was given extra attention in the following interviews and subsequently scrutinised in the data analysis. Recordings were transcribed using InqScribe software and MS Word in Swedish, and only excerpts were translated into English for demonstrative purposes. Data was coded inductively on thematic topics, initially highlighting broad organisational codes that reflected themes aligned with the study goals. These were highlighted on the transcript materials and added to an MS Excel sheet; this process was repeated to identify more specific substantive codes that emerged consistently from participants answers. Hence, we included them in new themes, which we later compared to the literature, and iteratively re-read and grounded (Bryman, 2016; Maxwell, 2009). Specifically, after the initial analysis was carried out by the first author and the main findings were discussed with co-authors, we linked the topic of AHCVs in relation to factors as knowledge, information, conflicts and implementation.

After the data was analysed and during the early manuscript writing process, we reported back our preliminary results to the interviewees as a form of respondent validation (Maxwell, 2009). GI coordinators were contacted through email and provided with a summary of the main results written in a popular science style in Swedish. Feedback was welcomed on these main findings either in writing or through two online feedback sessions of 3 h set up circa one week later. Only one interviewee participated in one feedback session.

## Results

### Maps as a Source of Conflict

Regional GI planning aimed, among other aspects, to investigate, map, and describe a network of areas of high conservation values (AHCVs) across each county. Generally, GI coordinators from the County Administrative Boards (CABs) reported that this task resulted in being highly polemic and sometimes even problematic due to stakeholders’ reactions. When AHCV maps were not a main source of conflict, they served as a tool for reflecting on how conservation governance would be carried out in practice on their basis.

The coordinators explained that the overall reaction towards mapping AHCVs was negative, in particular from stakeholders representing landowners and forest owner associations. Based on these accounts, we found three intertwined causes behind the opposition: (1) a dogmatic antagonism to the concept of AHCV, (2) historical contexts and the fear of the creation of nature reserves, and (3) misunderstanding and misinformation.

The main argument by landowner organisations reported by interviewees was, in principle, concerns over property rights, in the sense that ownership and utilisation rights would be harmed by GI initiatives and severely threatened by mapping AHCVs. Coordinator 10, from a county without major self-reported conflicts, explained that landowner interest groups are normally against organisations that attempt to find and map ecological values (including both the state agencies and environmental non-governmental organisations). This opinion was reiterated across the country by each regional chapter of the national landowner organisation. Prevailing discourses at the national level and the core positions of interest groups tend to influence the regional discussions.

“When we sent out the action plan, we got different opinions from these [stakeholders] who had general views, like different groups in society who sort of had the same views on all action plans around Sweden. So, you could almost see that they are perhaps fundamentally opposed to the state being able to point things out.” – Coordinator 10

Although one key raison d’étre of landowners’ interest groups is precisely to lobby for the protection of property rights, coordinators framed the backlash as a result of the historical context or experiences of State regulation, giving way to fear and a lack of trust towards authorities.

“People are still afraid that [land] with high [conservation] values will be pointed out. Maybe on their land it can be like this, and then you put a dead man’s hand on those areas and then all development stops, and it stops productive forestry, or it stops, like, development for housing. One is afraid that this [the GI work/AHCVs] is a claim about land for the benefit of nature conservation.” – Coordinator 7

In this case, forest owners’ property rights seem to extend to a right of deciding “what can be known” and “be mapped” about their lands. Knowledge about resources, in this case ecological values, is by itself perceived as a threat to ownership. Knowing where those values are located could lead to the creation of reserves or proscriptions on certain management practices. This makes forest owners distrustful of the motives of the State and potential actions it would be able to take if a CAB becomes aware of the existence of important ecological values. Therefore, as accounted by the interviewees, avoiding the creation of maps becomes a way to secure owners’ land rights.

“It’s about the right of ownership. If you say: ‘Ok. If an authority, if the state, sees that this land contains high natural values and it wishes to go ahead with the creation of reserves or protected areas, or to regulate the use of the land in some way, or [to say] that this [land] is not suitable area for housing development or exploitation; then you make it difficult for the landowner.’ That is what [owners are] afraid of. I think that it is the basic reason. and then I feel that there is a suspicion towards the authorities. Maybe also that it will only be complicated in the future if there are more rules about what [owners] can do on their land and such.” – Coordinator 7

According to the GI coordinators, misunderstanding and misinformation about GI and the AHCV maps seemed to have played an important role in exacerbating forest sector stakeholders’ antagonism. Publications in local newspapers and “loud” voices of key players were reported examples of why stakeholders got an “incorrect understanding” of the concept. Moreover, coordinators considered their responsibility, and even capacities or lack thereof, to change opinions and explain the purpose and intention of AHCV maps to stakeholders.

“I think people have read too much in the newspapers that the AHCVs are the new woodland key habitats. People have been simply misinformed, and there we have not managed to go out enough and be clear that this is not as dangerous as they think.” – Coordinator 17

### “Neutral” Knowledge Through AHCV Maps

The interviewed coordinators reported that many stakeholders, e.g., forest associations, landowner organisations, forest industry, do not share the *coordinators’* understanding of AHCVs. Coordinators considered that the stakeholders, to a certain degree, ignore what they feel AHCVs *in fact* are. Hence, adequately informing stakeholders was suggested as a means to solve one cause of the underlying conflict. In other words, if stakeholders possessed the same formal knowledge as coordinators, conflicts could be easily avoided.

“[The conflict] has a lot to do with the fact that people may not have understood the meaning of the ahcv, what they are, what they are to be used for. But they interpret it as some kind of future area protection, which is not the case. We tried to be as clear as possible. Both when we talk to people, when we write answers to questions, when we produce a publication, so that we really always write what AHCVs are; what they can be used for.” – Coordinator 10

In general, coordinators maintained that AHCV maps are technical knowledge and that AHCVs should be simply considered an effort by the CAB to better understand what valuable nature that exists in each county. In the quote below, coordinator 10 explained the relevance of AHCVs to ownership rights and expressed their effort to avoid misunderstanding.

“They [AHCVs] do not mean a change in obligations or rights to the landowner, but it’s actually current legislation that applies. So, this does not affect it [the rights of owning] at all. And it’s nothing physical, no physical boundaries out in nature, but we have only produced a small heat map so that you can see [for example] here we have very concentrated values, so you want to concentrate your conservation measures to an area where there is already lots that can be preserved in the surroundings, so you have the help of the AHCV [maps]. And if you don’t think you have any use for the AHCV, then you don’t even have to look at them. It’s completely voluntary. It should be voluntary. We tried to be very, very clear about that.” – Coordinator 10

The perceived AHCVs neutrality or objectivity is central to the coordinators’ reasoning. The lack of a legal mandate to change management gave AHCV maps an aspect of neutrality: Maps are not connected to any new laws, acts, directives or regulating policy, hence the AHCVs can be seen as pure information. Together with the AHCVs’ non-regulatory nature, their neutrality was also supported by the prescribed methodology to build maps and present factual data.

The general reasoning of the coordinators can be summarised as follows: firstly, the data only show what is indeed present observable conditions; the map basically just reveals and does not change anything or have any implications per se. Making a map is simply describing the nature - describing a place. Maps are, in this case, produced by objective methods, using GIS technology that just shows what is out there.

Secondly, coordinators gave large weight to how the maps are made. The mapping methodology was developed through long discussions and followed protocols developed over time involving the CABs and the Swedish Environmental Protection Agency. Thus, the action of using a well-thought-out technical methodology should consequently mean that the subjectivity and potential biases of the mapper are successfully mitigated. In this case, the maps are considered an output of GIS models and geostatistical techniques, which are not influenced by human interpretation or arbitrary decision-making. In other cases, AHCV maps explicitly involve a human interpreter in the final phases of mapping. Coordinators viewed this supervision as an expert opinion or as an added component of the method, which was useful to increase the quality and provide information that would be missed otherwise.

“We had a density raster that we did with a network analysis of core value zones. With that, we took a kilometre distance between them, and over that, I built up a network, and then we could sort out big areas, so that we sort of put a general buffer on them of 500 m, if I remember. And then, I got a precursor to the AHCVs, and then we went in manually, and if there were large clusters that were reasonably close to each other, and depending on what was in the landscape between them, we could link them together to [define] AHCVs.” – Coordinator 18

### Knowledge with a Purpose

The coordinator’s framing of the construction of maps as impartial, objective knowledge, and the fact that the maps do not give any explicit authority to influence management, does not prevent coordinators from understanding the implication that the maps could support the environmental conservation task of the CAB or forest management by owners. Several coordinators expressed that the AHCV maps could be primarily used as a prioritisation tool to utilise State resources aimed at nature conservation more efficiently. Given that not all the land can be “set aside” for conservation, a spatial prioritisation could maximise the benefits of each action or each hectare of land that is managed for maintaining biodiversity purposes. Moreover, such information is potentially beneficial to the forest sector in supporting their own voluntary prioritisation. Coordinators, especially in northern CABs, mentioned how cooperation can be easier with large companies that already have major voluntary conservation initiatives, e.g., Sveaskog’s eco parks, Holmen’s knowledge forests, and SCA’s conservation parks. Yet, AHCV maps with landscape scales could have different impacts for a non-industrial private forest owner, as reflected by coordinator 17:

“You have to prioritise both the state’s resources and also forestry resources, and then it is easier for a large forestry company that has holdings over large areas to concentrate their nature conservation efforts to, for example, an AHCV, because they have several areas to choose from. But a private landowner may end up with their entire holdings in an AHCV, and another landowner may not have any in an AHCV.” – Coordinator 17

Besides prioritising areas for conservation, the cartographic materials can be used as a catalysing tool for dialogue between the stakeholders.

“And then I think it should be used in dialogue. I don’t think it should be used to force people, but if you use facts for a fact-based dialogue and visualise it with maps, then I think you can get very far.” – Coordinator 7

“…One also understands that a private landowner who may end up with his entire estate in an ahcv will be afraid [and say] that ‘Ok, now I have to take much more care than anyone else’ or ‘Now I have to protect the whole [estate], now they’ll want my whole area as a reserve.’ But that doesn’t have to be the case at all. Then we need to have a dialogue about what you [as forest owners] could imagine doing to increase the natural values of your property, within that AHCV, what would work well on your property, what can you contribute? You will not have to contribute more than anyone else, but if you want to, you are very welcome to do so; but we can make sure that it is the right measure that fits best in that landscape to provide maximum benefit [to nature/biodiversity].” – Coordinator 17

Coordinators also pointed out limitations to the usefulness of the AHCV maps for efficient nature conservation, namely, their lack of a strong legal basis and a broad scope without providing detailed information. These meant that mapping AHCVs was experienced more as a task of mapping just for the sake of mapping, as mandated by the Government, and not because they were going to be useful for environmental conservation or governance.

“We’re in the process of producing it now [AHCV maps] because it’s a requirement mostly from the Swedish Environmental Protection Agency. It says in our mission that we should do it, but we may not really see a huge purpose for these maps because, for example, our forest maps are very rough, so there are AHCVs over most of the county.” – Coordinator 14

“The AHCVs that have been developed are painted with a very broad brush, so it may not be possible [to say where] that line goes… Like, is that particular farm there [within] that AHCV… The degree of detail is not so [high] that you can say that ‘Here it is nice and [here] it is not’. So, when questions come from outside, you sometimes want to have a little more [base] under your feet to be able to give a correct answer.” – Coordinator 16

### Adaptations in Policy Implementation

Coordinators reflected on two main types of issues: *(a)* conflicts over what the maps show and what they are used for; and *(b)* opposition to forests being mapped overall. As a result, coordinators adopted diverse approaches to balance their official mandate and the concerns of the forest stakeholders. Although each CAB worked within its specific context, adaptations could be categorised in two main approaches: cartographic adaptation (linked to the content of the maps) or processual adaptation (linked to the idea of mapping and CAB’s authoritative knowledge) (Table 2). One adaptation does not preclude the other, and CABs adopted different strategies during different periods or occasionally applied both simultaneously.

**Table 2:** Types, actions and example quotes of adaptation approaches by GI coordinators

Type	Actions	Quote examples
Cartographic adaptation	Areas are represented with fuzzy, or broad boundaries, do not show clear-cut divisions.	“Then the boundaries are also deliberately made rather coarse and a bit blobby, so a bit, not quite sharply after these limits on density or exactly according to the core values, but this is well precisely because you want to get this landscape thinking in. And that you want to show that within this landscape, we think we should work with pine forests, forest pine values. And it’s not just in this pocket, but it’s in this landscape, and exactly where that line is not clear, but it’s a bit diffuse. And it also has to do with the fact that it should not feel as if the authorities are now drawing a new border, and it goes exactly here.” – Coordinator 9
Processual adaptation	More dialogue and improved communication	“… We ended up realising that this was not going to be good, so we backed off then, and said on the spot that ‘OK, we are not publishing these documents now. So obviously, it raises too much emotion; we need to take a step back. Start again some dialogue meetings, communication efforts and so on.” – Coordinator 17
	Delay or withdrawal	“Our preliminary plan is that we hope that the debate on forests will feel a little calmer, that the green infrastructure issue has been settled, that people understand what it means, including landowners, so that we do not have to have a consultation on the AHCVs, but that we can present a finished proposal. So that we produce ‘This is what the value areas for forests in [the X County] look like, here you go, it’s a basis for working’, that’s what we want.” – Coordinator 13

*Cartographic adaptation* refers to the modifications made to how information is represented on the maps. Decisions on how to draw a map did not necessarily result as a direct consequence of a communicated opposition, as decisions might have been taken earlier, following the mapping methodologies. Yet, coordinators reflected on the importance of showing the AHCVs for stakeholders’ acceptance. The main goal was therefore that the AHCVs would be portrayed more as an indication than as a binary alternative between valuable versus not valuable areas. These adaptations mainly followed the argument that the main goal of the AHCVs is to show the overall location on the landscape and not to demarcate exact limits. Hence, delimitations of borders can be seen as a rough approximation on the scale at which they are mapped, not allowing inference of what properties are inside or outside of the AHCV.

*Processual adaptation* refers to the adjustments made to the collaborative governance process: either to (a) obtain a better comprehension of the AHCVs through increased dialogs or changing the tone of the task by putting greater emphasis on the voluntary nature of GI; or (b) by delaying or withdrawing the publication of the maps until opposition softens or the maps gain support from the CAB’s leadership. After seeing how problems arose in certain counties, few CABs even considered avoiding creating the maps altogether.

## Discussion

### Between a Rock and a Hard Place: Coordinators’ Awkward Position

One of the main insights of our study is the cumbersome task and the difficult position that GI coordinators found themselves in while developing forest AHCV maps. GI coordinators across Sweden had to direct the execution of a complex process that intersects diverging interests. By producing maps that highlight biodiversity-rich ecosystems, GI coordinators reported that their work was seen as conflicting with the interests of groups that advocate for productive forestry and private ownership rights. Additionally, GI coordinators are part of the CAB, an organisation that, due to its mandate to promote nature conservation, holds a reputation, among forest stakeholders, of a potentially *dangerous* authority that may overrule owners. Simultaneously, GI coordinators possessed no means of coercion but rather emphasised collaboration, a principal approach of Swedish GI policy (von Post et al., 2023). It is in this context that the AHCV maps are caught in a peculiar dynamic, balancing the customary aim of explicit spatial delineation with efforts to prevent conflict by *blurring* the boundaries through different adaptation approaches. Thus, the idea of the mapping - and its embedded authoritative knowledge (Pynnönen, 2020) - leads to the need for processual adaptations; while the content and purpose of the map itself for environmental planning (Tsouvalis et al., 2000), leads to the cartographic adaptations.

Coordinators generally had a natural science background, with a third-level education in the fields of biology, conservation biology or ecology. This type of scientific training normally reinforces the ontological and epistemological views of knowledge as neutral to the observer (Latour, 1988). This perception is likely reinforced by a key principle of public administration: that of impartiality. On the other hand, coordinators’ position within the formal policy process is one with a degree of autonomy and discretion in the implementation of administrative law and policy provided by their executive agencies, departments or ministries. The degree of this autonomy can vary and is institutionalised, but critical for the formation of public policy (Carelli & Peters, 2024). Officials may often be shielded from experiencing the politics of policymaking while exercising this autonomy, but this must fall away when processes include direct public participation in its variety of forms. Participatory environmental governance is a key feature of environmental policy-making in Sweden and is often demanded by both legislative and administrative law (Yin, 2010). Participatory governance is inherently political and represents an attempt to re-embed democratic and deliberative traditions into administrative approaches to policymaking. The politicisation of map-making in our case would appear to occur in this juncture.

Deliberations about the utility of the maps became the central discussion point when the production of knowledge via this tool was used to support the CABs’ nature conservation goal. For whom are these maps useful, and how might they be used? The need to address these questions certainly makes it difficult for coordinators to be seen as neutral by other stakeholders. Limited exposure and inherent complexities of social science theories of knowledge construction make them often inaccessible in natural science training (Moon & Blackman, 2014). Consequently, such a lack of appropriate tools to solve human dimension problems (e.g., as different values and perceptions) limits the capacity of conservation work (Bennett et al., 2017). In the AHCV case, authorities perceived maps as only uncovering preexisting natural values, as representing only pure knowledge and being harmless because of their voluntary nature; perhaps leading to higher chances of conflicts when missing a priori the understanding of other actors’ views. Ultimately, the coordinators’ onto-epistemological framings of themselves failed to recognise how mapping functions as a form of power (Whitehead et al., 2007), increasing the visibility and legibility of certain phenomena as valuable. Hence, a wider acknowledgement of the social construction of knowledge and related power dynamics could provide more nuanced discussions around map representations and meanings (Straume, 2014), and improve the effectiveness and legitimacy of such conservation initiatives (Bennett et al., 2017).

### A Criticised Policy Tool Within a Heated Debate

Another main insight from our study is how undertaking the regional GI planning and mapping of AHCVs created tensions due to unsolved land use trade-off challenges. Based on a policy analysis, von Post et al. (2023) foresaw limitations on the success of Swedish GI policy. They found that this novel GI policy stems from a policy assemblage of a number of EU and national regulations. Such an assemblage tries to merge diverse perspectives on landscape approaches and ecosystem services yet fails to acknowledge the underlying need for trade-offs in land use if biodiversity conservation is to take place. Our results, highlighting the tensions reported by GI coordinators, help to validate the important critique of the policy design drawn by von Post et al. (2023). Furthermore, in their own reflections, coordinators problematised the policy instrument due to its voluntary nature, as maps are not legally binding and do not limit owners’ management; and despite being intended as an informational policy instrument, its lack of clarity in terms of scales, detail-level, and usefulness for decision-making makes the policy difficult to implement and potential impacts hard to foresee. Finally, implementation of GI plans was not followed by specific earmarked funding to compensate losses to landowners willing to set-aside forest, which could have contributed to the causes of its seldom use (Karlsson et al., 2024) or punctual implementation across the country (Naturvårdsverket, 2023).

Moreover, we found that a history of conflict relating to mapping was a clear factor influencing the tone surrounding AHCVs. Coordinators felt that the AHCVs were perceived as a new variant of the previously contested woodland key habitats framework (Götmark, 2009). Negative experiences can lead to disregard or hinder new approaches that the CABs might propose. After examining the participatory process of GI planning in Scania county, Karlsson et al. (2024) also noted the importance of history in the GI collaborative governance processes, making the previous experiences a main factor for taking a leap of faith to engage in new collaborations. Despite Karlsson et al., (2024) multi-stakeholder description of the GI process being limited to one county and not necessarily generalisable to whole Sweden, we found through the perception of coordinators that the GI process showed a similar pattern across the country. The GI planning process is now finalised, but future studies that look at GI implementation could benefit from examining perceptions of multiple stakeholders, including forest managers and owners. The dogmatic and conflicting positionality in the conservation versus production discourse hindered the CABs’ task in GI planning. These tensions reflect previously studied Swedish forest conflicts (Jakobsson et al., 2021). Participants reported that the argument of eroding property rights was key for the opposition, put forward and driven by like-minded stakeholders present across the country, in particular by the Federation of Swedish Farmers. The perception of a homogeneous response orchestrated by the central headquarters of a powerful stakeholder resonates with the ideological homogenisation and echo-chamber characteristic of the Swedish forest conflicts (Sténs & Mårald, 2020).

Although, ours is a Swedish case, existing knowledge systems are also embedded in larger political dynamics of forest governance. EU forest policy has in comparison to other sectors (environment or agriculture) largely remained national and followed the subsidiary principle. Yet, as new policies such as the EU Green Infrastructure Strategy, or the EU Forest strategy, make their way into national policy (von Post et al., 2023); these top-down “one-size-fits-all” policies and their inherent knowledge models could predictably lead to confrontations (Sandström et al., 2022).

### Talking About Knowledge to Improve Nature Governance

The use of maps is ubiquitous for the professionals who plan and manage forest operations, as well as for the public officials working with nature conservation; understanding how stakeholders with decision-making power see this information will be ever more crucial for collaborative governance. Furthermore, the amount of information about forest ecosystems increases ever more rapidly, not least due to remote sensing data aided by artificial intelligence techniques (Nitoslawski et al., 2021). Similarly, multiple research tracks on the natural dimensions of green infrastructure (See also Liquete et al., 2015; Mikusiński et al., 2021; Snäll et al., 2016) are extremely valuable and should be expanded given the current needs for global biodiversity conservation (IPBES, 2019). However, it remains to be seen if an abundance of cartographic information will bring better policy legitimacy and stakeholder acceptance. Positive examples from Finnish cases show that participatory spatial prioritisation and actual inclusion of local knowledge can help to align mapping outcomes with stakeholder values (Kareksela et al., 2020; Paloniemi et al., 2018; Salomaa et al., 2016). Understanding how different stakeholders perceive the meaning and role of maps will be crucial if collaborative governance is to remain a key policy instrument in forestry and natural resources management (Bjärstig et al., 2024). To improve environmental management, it is essential to involve stakeholders and decision-makers early and actively re-anchor existing solutions with them. Hence, the necessary time and resources to accommodate this must be included in the proposed process. Coordinators’ own strategies, in particular the *processual adaptation* including delay and withdrawal, were deemed viable options to bridge and improve understanding between stakeholders. Implementing frameworks that promote knowledge co-production, such as knowledge governance (Gerritsen et al., 2013), can enable more successful outcomes for participatory processes in environmental management.

## Conclusion

County coordinators of GI plans had a predominant view that maps constitute a neutral and objective instrument for spatial approximation of ecologically valuable areas. However, our interviews unequivocally reveal that a map is much more than a polygon that mechanically optimises the spatial allocation. In somewhat of a contradiction, the coordinators admit that maps may affect the allocation of priorities and resources, even if on a supposedly voluntary basis. Given this acknowledgement, it is perhaps less surprising that the AHCV maps turned out to be one of the most sensitive battling arenas within the GI plans in many counties investigated. GI planning and coordinators’ work were from the onset extremely difficult due to the atmosphere of conflict around forest resources, unsolved land use trade-offs and previous experiences of forest stakeholders, influencing coordinators’ remedy strategies, including cartographic and processual adaptations. Our analysis unequivocally reveals that mapping is not merely a technical exercise, but rather an institutionally conditioned and socially contested output of governance.

Given the urgent need to improve biodiversity conservation in Swedish forests, coupled with limited resources for implementation, explicit spatial prioritisation of GI is not only warranted but necessary. Nevertheless, if conservation policies in private forest rely primarily on voluntarism, there is a clear need for better strategies of mapping, together with better strategies for communication, administration and resource allocation. These should begin with an open acknowledgement that maps are not neutral instruments but instead play an active role in shaping power dynamics and stakeholder relations within forested landscapes.

## Data Availability

No datasets were generated or analysed during the current study.
